# Recovery Profiles After MOAA/S-Guided Remifentanil- Versus Fentanyl-Based Propofol Sedation for Gastrointestinal Endoscopy: An IPTW-Adjusted Retrospective Cohort Study

**DOI:** 10.3390/jcm15187276

**Published:** 2026-09-19

**Authors:** Ahmet Salih Tüzen, Rıdvan Özlük, Ayşe Rumeysa Kocatürk, Mehmet Ali Coşar, Zülfükar Bilge, Birzat Emre Gölboyu, Sezgin Vatansever, Murat Aksun

**Affiliations:** 1 Department of Anesthesiology and Reanimation, Izmir Katip Celebi University Atatürk Training and Research Hospital, 35360 Izmir, Türkiye; ozluk45564@gmail.com (R.Ö.); armysa254@gmail.com (A.R.K.); malicosar@gmail.com (M.A.C.); birzatemre@windowslive.com (B.E.G.); murataksun@yahoo.com (M.A.); 2 Department of Physiology, Graduate School of Health Sciences, Dokuz Eylül University, 35330 Izmir, Türkiye; 3 Department of Gastroenterology, Izmir Katip Celebi University Atatürk Training and Research Hospital, 35360 Izmir, Türkiye; zulfukarbilge@mynet.com (Z.B.); sezgin.vatansever@ikcu.edu.tr (S.V.); 4 Department of Intensive Care, Izmir Katip Celebi University Atatürk Training and Research Hospital, 35360 Izmir, Türkiye

**Keywords:** fentanyl, gastrointestinal endoscopy, post-anesthesia care unit, propofol, recovery time, remifentanil

## Abstract

**Background/Objectives:** In high-volume gastrointestinal (GI) endoscopy units, the choice of opioid adjunct may influence awakening, post-anesthesia care unit (PACU) utilization, concomitant sedative requirements, and early cardiorespiratory events. Remifentanil has a rapid and predictable offset, whereas fentanyl may produce more prolonged residual effects. We compared postanesthetic recovery after remifentanil- versus fentanyl-based propofol sedation in routine clinical practice. **Methods:** This single-center retrospective cohort study included adults undergoing elective upper GI endoscopy, colonoscopy, or combined procedures under anesthesiologist-directed propofol-based sedation between 1 July 2025 and 31 January 2026. Sedation was administered according to an institutional protocol targeting a Modified Observer’s Assessment of Alertness/Sedation (MOAA/S) score of 2–3. Patients received either remifentanil or fentanyl as the opioid adjunct. The primary outcome was the interval from procedure completion to the first documented response to verbal command or purposeful awakening. Secondary outcomes included procedure and anesthesia durations, PACU length of stay, propofol and midazolam doses, and early respiratory and hemodynamic events. Stabilized inverse probability of treatment weighting (IPTW) was used to balance measured baseline characteristics. **Results:** Among 545 patients, 280 received fentanyl and 265 received remifentanil. All assessed covariates achieved acceptable balance after weighting. Median MOAA/S score was 2 [2-2] in both groups before and after IPTW adjustment, and target-range, deeper-than-target, and lighter-than-target MOAA/S categories did not differ significantly between groups. Weighted mean recovery time was shorter with remifentanil than with fentanyl (22.6 vs. 34.1 s; adjusted mean difference, −11.4 s; 95% CI, −13.4 to −9.5; *p* < 0.001). Remifentanil-based sedation was also associated with shorter procedure and anesthesia durations, shorter PACU length of stay, and lower propofol and midazolam doses; these findings remained significant after false discovery rate (FDR) adjustment. No respiratory, hemodynamic, or PACU adverse outcome differed significantly after multiplicity adjustment. **Conclusions:** Remifentanil-based propofol sedation was associated with shorter time to first purposeful response, shorter PACU stay, and lower concomitant sedative exposure than fentanyl-based sedation during routine GI endoscopy. Documented MOAA/S findings did not show a higher frequency of lighter-than-target clinically assessed sedation in the remifentanil group. These findings may reflect the combined contribution of remifentanil’s rapid offset and lower concomitant sedative exposure, but they should be interpreted as associations within a clinician-directed sedation strategy. The retrospective, nonrandomized design precludes causal or safety-equivalence conclusions.

## 1. Introduction

Gastrointestinal (GI) endoscopy frequently requires sedation to improve patient comfort, facilitate procedural completion, and maintain acceptable operating conditions. In high-volume endoscopy units, an effective sedation strategy must provide adequate hypnosis and analgesia while permitting rapid recovery and safe discharge. Propofol-based sedation is widely used because of its rapid onset and relatively short duration of action; however, its dose-dependent respiratory and hemodynamic effects often necessitate the addition of an opioid to reduce hypnotic requirements and suppress responses to procedural stimulation [1,2,3].

Fentanyl is commonly combined with propofol because of its potent analgesic effect and familiarity in procedural sedation. Nevertheless, its residual effects may contribute to delayed awakening and prolonged postprocedural monitoring, particularly when it is combined with propofol or other sedatives [4,5,6,7]. In contrast, remifentanil is an ultra-short-acting μ-opioid receptor agonist with rapid titratability and offset. In direct endoscopic comparisons, remifentanil-based propofol sedation has been associated with reduced propofol requirements and faster recovery than fentanyl-based regimens [4,8,9]. However, the rapid onset and potent respiratory depressant effect of remifentanil require careful dose titration and continuous cardiorespiratory monitoring, as apnea may occur more frequently with some dosing strategies [4,10].

Recovery after endoscopic sedation is clinically and operationally relevant. Delayed awakening and prolonged post-anesthesia care unit (PACU) stay may impair patient throughput, increase staffing and monitoring requirements, and postpone discharge. Recovery is influenced not only by opioid selection but also by propofol and benzodiazepine exposure, procedure duration, patient characteristics, and institutional workflow [2,5,11]. Randomized studies of upper GI endoscopy and colonoscopy have shown that remifentanil-containing propofol regimens may shorten awakening or recovery and reduce hypnotic requirements [12,13]. Nevertheless, remifentanil-propofol sedation has been associated with apnea and increased airway-rescue requirements in some studies, underscoring the importance of careful dose titration and continuous respiratory monitoring [4,14]. Evidence directly comparing remifentanil- and fentanyl-based propofol sedation under routine clinical conditions remains limited and heterogeneous.

The primary objective of this retrospective cohort study was to compare postanesthetic recovery time between remifentanil- and fentanyl-based propofol sedation in adults undergoing routine GI endoscopy. Secondary objectives were to compare procedure and anesthesia durations, PACU length of stay, propofol and midazolam requirements, and early respiratory, hemodynamic, and PACU adverse outcomes. We hypothesized that remifentanil-based sedation would be associated with shorter time to first purposeful response, shorter PACU stay, and lower concomitant sedative exposure, without a statistically significant increase in recorded early cardiorespiratory or PACU adverse events.

## 2. Materials and Methods

### 2.1. Study Design, Setting and Ethical Approval

This single-center retrospective cohort study was conducted in the Endoscopy Unit of Izmir Katip Celebi University Atatürk Training and Research Hospital, a tertiary academic referral center in Izmir, Türkiye. The source population comprised adults undergoing elective upper GI endoscopy, colonoscopy, or both procedures during the same sedation session under anesthesiologist-directed propofol-based sedation from 1 July 2025 through 31 January 2026. All procedural and sedation management was delivered according to routine institutional practice, and no aspect of patient care was modified for research purposes.

The study protocol was approved by the Izmir Katip Celebi University Atatürk Training and Research Hospital Health Sciences Ethics Committee (approval no. 0115; 12 February 2026). The ethics committee waived the requirement for written informed consent because the study involved the retrospective analysis of de-identified clinical data collected during routine care. The study was conducted in accordance with the Declaration of Helsinki and reported in accordance with the Strengthening the Reporting of Observational Studies in Epidemiology (STROBE) statement for cohort studies [15].

### 2.2. Study Population and Exposure Definition

Patients were eligible if they were aged ≥ 18 years, underwent upper GI endoscopy, colonoscopy, or a combined procedure under anesthesiologist-directed sedation, received propofol-based sedation with either fentanyl or remifentanil, were transferred to the PACU after the procedure, and had complete documentation of opioid exposure, recovery time, procedural and anesthesia timestamps, PACU admission and discharge times, anesthetic drug doses, baseline covariates, and defined safety outcomes.

The exclusion criteria were general anesthesia, endotracheal intubation, or other advanced airway instrumentation; emergency endoscopy for a life-threatening indication; and advanced, specialized, or higher-risk endoscopic procedures conducted through a different anesthetic pathway, including endoscopic ultrasound, endoscopic retrograde cholangiopancreatography, peroral endoscopic myotomy, percutaneous endoscopic gastrostomy, and endoscopic submucosal dissection. Procedures were also excluded when both study opioids or neither study opioid had been administered, when additional anesthetic techniques outside the routine sedation pathway were used, when PACU follow-up was unavailable, or when data required for exposure classification, outcome assessment, or propensity score estimation were incomplete. The exposure was defined according to the opioid documented in the anesthesia record and categorized as remifentanil-based or fentanyl-based sedation. Opioid selection was not randomized and reflected the clinical judgment of the attending anesthesiologist. Patient identifiers were cross-checked to ensure that each patient contributed only one eligible procedure, such that the patient and procedure represented the same independent unit of analysis.

### 2.3. Endoscopic Procedures and Sedation Management

Upper GI endoscopy was defined as flexible endoscopic examination of the esophagus, stomach, and duodenum through the oral route. Colonoscopy was defined as flexible endoscopic examination of the colon, with or without terminal ileal intubation. A combined procedure comprised sequential upper GI endoscopy and colonoscopy performed during the same sedation session. Endoscopic procedures were performed by the same gastroenterology team, consisting of three specialist gastroenterologists working in the endoscopy unit. Thus, both opioid groups were managed within the same procedural environment and by endoscopists with comparable specialist-level clinical experience.

Sedation was administered according to the institutional procedural sedation protocol by the same anesthesia team, consisting of two attending anesthesiologists, two anesthesiology residents, and three anesthesia nurses. The attending anesthesiologists were experienced in the pharmacodynamic profiles, titration characteristics, and potential respiratory and hemodynamic adverse effects of both fentanyl and remifentanil. Both opioid strategies were delivered by clinicians working within the same institutional sedation pathway and with comparable team-level experience. Sedation depth was assessed clinically using the Modified Observer’s Assessment of Alertness/Sedation (MOAA/S) scale, with a usual target of moderate-to-deep sedation corresponding to a score of 2–3 while maintaining spontaneous ventilation. Within this protocol, sedative and opioid doses were titrated to achieve procedural tolerance at the target clinical sedation depth rather than according to fixed dose targets. Procedural tolerance was operationally defined as suppression of excessive movement, discomfort, or responsiveness that interfered with endoscopic completion, while remaining within predefined respiratory and hemodynamic guardrails. Respiratory guardrails for further dose escalation included preserved spontaneous ventilation, absence of clinically or capnographically detected apnea, no requirement for manual airway support, and maintenance of SpO_2_ ≥ 90%. Hemodynamic guardrails included systolic arterial pressure ≥ 90 mmHg, mean arterial pressure ≥ 65 mmHg, absence of a >20% decrease from baseline arterial pressure, heart rate ≥ 50 beats/min, and no immediate requirement for vasopressor treatment or intravenous crystalloid bolus administration. Intravenous lidocaine (Aritmal 2%, Osel, Istanbul, Turkey) was administered as a 0.5 mg/kg bolus at the start of sedation. Propofol (Propofol 1%, Polifarma, Istanbul, Turkey) was administered intravenously in titrated boluses. The opioid component consisted of either remifentanil (Rentanil, VEM Ilac, Istanbul, Turkey) or fentanyl (Fentanyl-PF, Polifarma, Istanbul, Turkey). For upper GI endoscopy, remifentanil was generally administered as a titrated infusion of 0.025–0.05 µg/kg/min or as titrated boluses, whereas fentanyl was generally administered in intermittent boluses of 0.5–1.0 µg/kg. For colonoscopy and combined procedures, remifentanil was generally administered at 0.05–0.10 µg/kg/min or as titrated boluses, whereas fentanyl was generally administered as an initial bolus of 1.0–1.5 µg/kg with additional boluses at intervals of at least 15 min when clinically indicated. Midazolam (Zolamid, VEM Ilac, Istanbul, Turkey) at approximately 0.01–0.02 mg/kg was used intravenously as an adjunct in the routine sedation pathway for colonoscopy and combined procedures. Additional sedative or opioid dosing was guided by real-time clinical assessment of procedural stimulation, patient movement, increased responsiveness, discomfort, respiratory and hemodynamic guardrails.

Although the same anesthesia team managed both opioid strategies, drug titration was not protocol-fixed for research purposes and reflected real-time clinical assessment, procedural stimulation, patient responsiveness, and cardiorespiratory conditions. Drug administration was documented contemporaneously in the anesthesia record and abstracted as cumulative intra-procedural doses for analysis. Initial opioid administration occurred at the start of sedation before or during endoscope insertion, whereas subsequent propofol, opioid, or midazolam dosing was administered according to procedural stimulation, movement, increased responsiveness, discomfort, and cardiorespiratory tolerance. However, detailed time-stamped rescue-dosing trajectories were not uniformly available in the structured dataset; therefore, the analysis focused on cumulative administered doses rather than dose-timing patterns. During sedation, all patients received supplemental oxygen at a flow rate of 6 L/min. Standard intra-procedural monitoring comprised continuous electrocardiography, pulse oximetry, and capnography for all included cases, together with noninvasive blood-pressure measurements at 3 min intervals. Capnography was used clinically for respiratory monitoring and apnea detection; however, detailed capnographic waveform characteristics and event-level capnography timing were not consistently available for retrospective extraction. Airway support, vasopressor treatment, and intravenous crystalloid boluses were provided according to routine clinical practice, when required. After the procedure, all patients were transferred to the PACU for pulse oximetry, noninvasive blood pressure and heart rate monitoring, and serial assessment of consciousness, ventilation, pain, nausea or vomiting, and hemodynamic stability. Recovery was supervised by the PACU nursing staff in collaboration with the attending anesthesiologist. Discharge readiness was assessed using the Modified Aldrete Score. Patients were discharged when a score of at least 9 was achieved, together with stable vital signs, adequate spontaneous ventilation and oxygenation, acceptable pain and nausea control, and no unresolved respiratory or hemodynamic complications.

### 2.4. Data Sources and Variables

Data were obtained from electronic anesthesia records, endoscopy reports, and PACU documentation. Baseline demographic and clinical characteristics were abstracted from the preanesthetic assessment and electronic medical records, whereas procedural timestamps, anesthetic drug administration, intra-procedural events, and recovery data were obtained from the corresponding anesthesia and PACU records. For medication exposure, cumulative administered doses were available for analysis, whereas detailed time-stamped dose-by-dose rescue administration patterns were not uniformly extractable from the structured records. Data were entered into a standardized study database after the removal of direct patient identifiers.

The prespecified baseline covariates were age, sex, American Society of Anesthesiologists (ASA) physical status, procedure type, smoking history, suspected or diagnosed obstructive sleep apnea syndrome (OSAS), chronic obstructive pulmonary disease (COPD) or asthma, cardiovascular disease, hypertension, diabetes mellitus, chronic kidney disease, chronic liver disease, neurological disease, and anticoagulant or antiplatelet therapy. These variables were included as pre-exposure characteristics potentially associated with opioid selection and recovery outcomes.

Procedure duration was defined as the interval from endoscope insertion to endoscope withdrawal. Anesthesia duration was defined as the interval between the documented start and end times of anesthesia care. PACU length of stay was defined as the interval from documented PACU admission to formal discharge. Documented MOAA/S score was extracted from anesthesia records as a clinical measure of achieved sedation depth. Medication variables comprised the cumulative doses of propofol, midazolam, and the assigned opioid administered during the sedation episode. Because fentanyl and remifentanil doses are not directly pharmacologically equivalent, the assigned opioid dose was summarized descriptively and was not subjected to a between-group inferential comparison.

### 2.5. Outcome Measures

The primary outcome was postanesthetic recovery time, defined as the interval in seconds from the completion of the endoscopic procedure to the first documented response to verbal command or purposeful awakening in the anesthesia record. This endpoint was intended to capture early return of responsiveness and should not be interpreted as complete cognitive recovery, psychomotor recovery, or discharge readiness. Continuous secondary outcomes included procedure duration, anesthesia duration, PACU length of stay, cumulative propofol dose, and cumulative midazolam dose. These outcomes were evaluated as absolute between-group differences. The cumulative dose of the assigned opioid was reported descriptively. Sedation-depth comparability variables included documented MOAA/S score, target-range sedation, deeper-than-target sedation, and lighter-than-target sedation. The institutional target range was defined as MOAA/S 2–3. MOAA/S ≤ 1 was classified as deeper-than-target sedation, whereas MOAA/S ≥ 4 was classified as lighter-than-target sedation.

The same respiratory and hemodynamic parameters used as clinical guardrails during sedation titration were used to define recorded safety outcomes. Respiratory safety outcomes included apnea, requirement for an airway maneuver, and peripheral oxygen saturation (SpO_2_) < 90%. Apnea was defined as a clinician-documented cessation of spontaneous respiratory activity identified by capnographic and clinical monitoring that prompted stimulation, modification or interruption of sedative administration, or airway support. Airway maneuvers included jaw thrust, chin lift, or other manual interventions performed to maintain airway patency. Oxygen desaturation was defined as any documented SpO_2_ value < 90% during the procedure or PACU observation. Hemodynamic outcomes included hypotension, bradycardia, vasopressor administration, and intravenous crystalloid bolus administration. Hypotension was defined as a systolic arterial pressure < 90 mmHg, a mean arterial pressure < 65 mmHg, or a decrease of >20% from the baseline. Bradycardia was defined as a heart rate < 50 beats/min. A composite hemodynamic event or intervention was defined as the occurrence of hypotension, bradycardia, vasopressor administration, or intravenous crystalloid bolus administration during the periprocedural period. PACU adverse outcomes included documented nausea or vomiting and pain requiring rescue analgesic treatment. Respiratory, hemodynamic, and PACU outcomes were not mutually exclusive; therefore, an individual patient could contribute to more than one outcome category. All respiratory, hemodynamic, and PACU adverse outcomes were ascertained retrospectively from contemporaneous anesthesia and PACU records, without independent event adjudication. These variables captured recorded event occurrence only. Event-level details, including duration, severity, exact timing, escalation level, and response to intervention, were not consistently available in the structured dataset.

### 2.6. Statistical Analysis

Analyses were performed using RStudio (version 2025.09.2+418). The distributions of continuous variables were assessed using histograms and Q-Q plots. Continuous variables with normal distributions were summarized as mean ± standard deviation, whereas skewed or ordinal variables were summarized as median [25th–75th percentile]. Categorical variables were presented as n (%). In crude comparisons, continuous variables were compared using Welch’s independent-samples *t*-test, skewed or ordinal variables using the Mann–Whitney U test, and categorical variables using the chi-square test or Fisher’s exact test, as appropriate. Crude effect estimates were reported as mean differences, Hodges–Lehmann median differences, or absolute percentage-point (pp) differences, each with 95% confidence intervals (CIs). For multilevel categorical variables, *p*-values represented global comparisons, whereas CIs represented level-specific one-versus-rest contrasts. Missing data in key analytic fields were reviewed during data verification. After verification of the dataset, no additional records were excluded because of unresolved missing data, and all analyses were conducted on the same final analytic cohort of 545 patients.

To account for measured confounding, propensity scores for receiving remifentanil-based rather than fentanyl-based sedation were estimated using multivariable logistic regression. The propensity-score model included pre-exposure demographic, procedural, comorbidity, and medication-history variables considered potentially associated with opioid selection or postanesthetic recovery: age, sex, ASA physical status, procedure type, smoking history, suspected or diagnosed OSAS, COPD or asthma, cardiovascular disease, hypertension, diabetes mellitus, chronic kidney disease, chronic liver disease, neurological disease, and anticoagulant or antiplatelet therapy. Stabilized inverse probability of treatment weights (IPTWs) were calculated to estimate average treatment effects. Postexposure variables, including achieved sedation-depth measures, procedure duration, anesthesia duration, PACU length of stay, and administered sedative doses, were not included in the propensity-score model. Propensity-score overlap and weight distributions were assessed graphically, and covariate balance was evaluated using absolute standardized mean differences, with values < 0.10 considered acceptable (Supplementary Figure S1). For multilevel variables, the largest level-specific standardized mean difference was reported. No weight trimming or truncation was applied, and effective sample sizes were calculated after weighting.

Crude and IPTW-weighted analyses were then performed according to variable type. The primary outcome and continuous secondary outcomes were analyzed using weighted linear models with stabilized IPTWs and robust standard errors and were reported as adjusted mean differences. The primary outcome was additionally evaluated using a log-transformed weighted model and reported as a geometric mean ratio. Ordinal sedation-depth variables were analyzed using rank-based methods, whereas binary sedation-depth and safety outcomes were analyzed using weighted linear probability models and reported as adjusted absolute risk differences. Multiplicity was controlled using the Benjamini–Hochberg false discovery rate (FDR) procedure, applied separately to secondary procedural/sedation-depth/drug-exposure outcomes and to binary safety outcomes. The assigned opioid dose was excluded from inferential testing and multiplicity adjustment because it was reported descriptively only. Procedure-type subgroup effects were assessed using an IPTW-weighted interaction model. A further sensitivity analysis used conventional multivariable linear regression with HC3 robust standard errors and the same baseline covariates included in the propensity-score model. Effect estimates were expressed as remifentanil minus fentanyl. All tests were two-sided; *p* < 0.05 was considered significant for the primary outcome, and FDR-adjusted *p* < 0.05 was used for secondary outcomes.

Sample size calculation was based on preliminary data for the primary outcome. The preliminary analysis indicated a mean postanesthetic recovery time of 28 s, with a standard deviation of 11 s. A 10% reduction in recovery time in the remifentanil group, corresponding to an absolute difference of 2.8 s and a standardized mean difference of approximately 0.255, was selected a priori as a conservative minimum detectable difference for study planning rather than as a definitive threshold for patient-level clinical importance. Assuming a two-sided alpha level of 0.05 and 80% statistical power, the minimum analyzable sample size was calculated as 240 patients per group. After allowing for a 10% rate of incomplete records, exclusions, or unavailable outcome data, the minimum target sample size was increased to 264 patients per group.

## 3. Results

**Figure 1:**
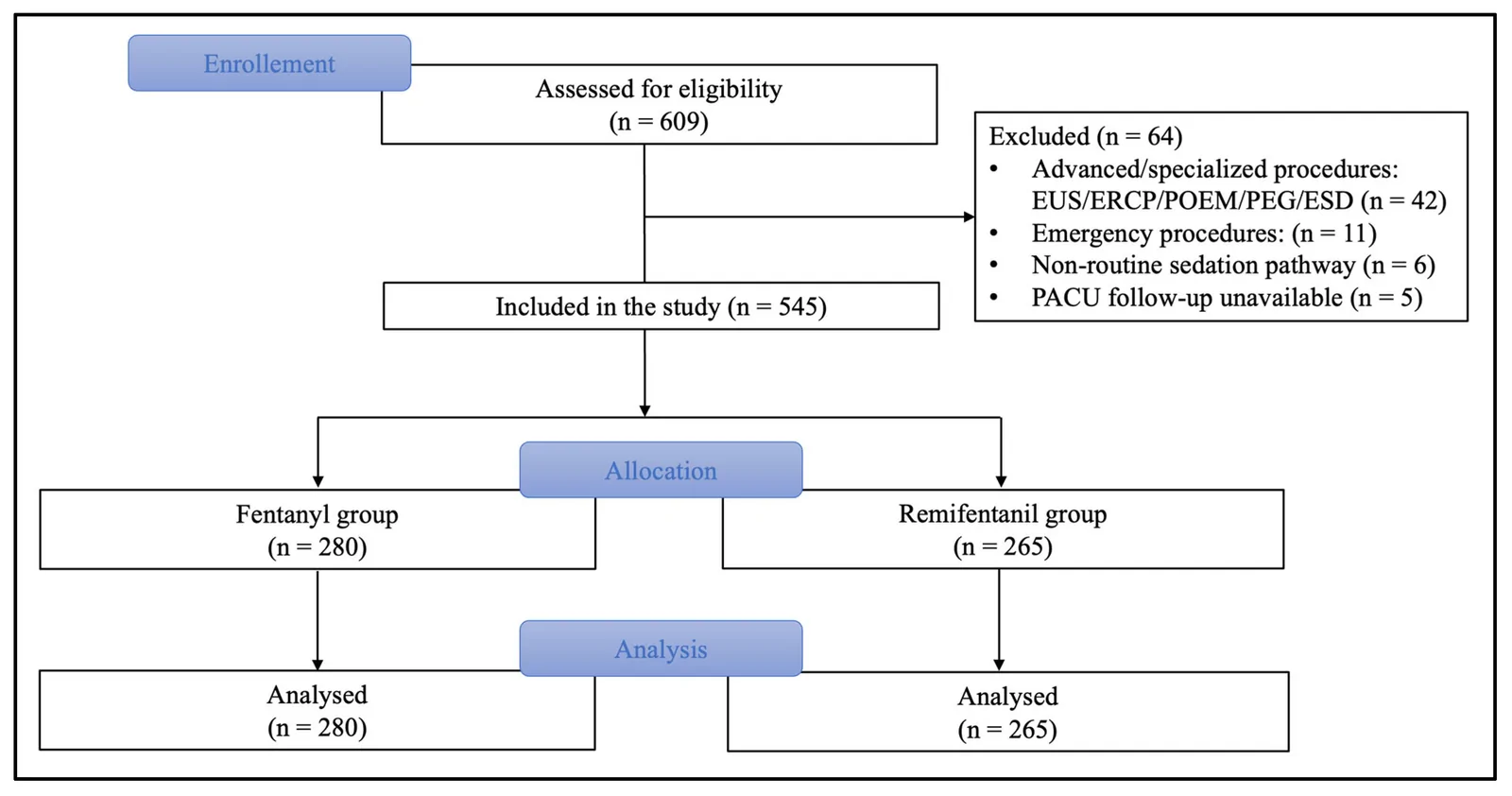
STROBE flow diagram of participant selection.

During the study period, 609 adult endoscopic sedation records were screened for eligibility. Of these, 42 records were excluded because they involved advanced or specialized endoscopic procedures, 11 because of emergency indications, 6 because additional anesthetic techniques outside the routine sedation pathway were used, and 5 because PACU follow-up was unavailable. A total of 545 patients, each contributing one independent GI endoscopic procedure, were included in the analysis; 280 received fentanyl-based sedation, and 265 received remifentanil-based sedation (Figure 1).

The demographic characteristics and ASA physical status were similar between the groups, with low unweighted SMDs for age, sex, and ASA distribution (Table 1). The procedure type distribution did not differ significantly between the groups, although a mild imbalance was observed according to the largest level-specific SMD. Among the baseline comorbidities, hypertension was less frequent in the remifentanil group than in the fentanyl group [37.7% vs. 47.5%; difference, −9.8 percentage points; 95% CI, −17.9 to −1.5; SMD = 0.198; *p* = 0.021]. Other comorbidities and medication-related characteristics showed no statistically significant between-group differences, although a mild unweighted SMD imbalance was observed for COPD/asthma, chronic kidney disease, chronic liver disease, and neurological disease (Table 1).

To account for the measured baseline imbalance between the opioid groups, propensity scores were estimated using demographic, clinical, comorbidity, medication history, and procedure type covariates. Because each patient contributed to one procedure, all 545 independent observations were retained for the IPTW analysis. The stabilized weights were well distributed, with a median of 0.963, mean of 1.002, and maximum of 3.253; no stabilized weight exceeded five. The effective sample size after weighting was 267.6 in the fentanyl group and 249.5 in the remifentanil group. Propensity score distributions demonstrated adequate overlap between groups, and the stabilized weight distribution did not indicate any influential extreme weights. After IPTW, all assessed covariates achieved an acceptable balance, with weighted SMDs < 0.10; the maximum SMD decreased from 0.198 before weighting to 0.021 after weighting (Table 2 and Supplementary Figure S1).

Postanesthetic recovery time, defined as the time to first documented response to verbal command or purposeful awakening, was shorter in the remifentanil group than in the fentanyl group in both crude and IPTW-adjusted analyses (Table 3). After IPTW adjustment, the weighted mean recovery time remained shorter in the remifentanil group than in the fentanyl group (22.6 s [95% CI, 21.2 to 24.0] vs. 34.1 s [95% CI, 32.8 to 35.3]), with an adjusted mean difference of −11.4 s [95% CI, −13.4 to −9.5; *p* < 0.001]. The log-transformed IPTW sensitivity analysis yielded a consistent relative effect estimate, with a geometric mean ratio of 0.628 [95% CI, 0.591 to 0.667; *p* < 0.001] (Table 3).

Documented sedation depth, secondary procedural time outcomes, and anesthetic drug exposure are shown in Table 4. Median MOAA/S score was 2 [2-2] in both groups before and after IPTW adjustment, with no significant between-group difference after FDR correction. The proportion of patients maintained within the institutional target MOAA/S range of 2–3 was similar between groups after IPTW adjustment [94.2% in the fentanyl group vs. 92.9% in the remifentanil group; adjusted risk difference, −1.3 percentage points; 95% CI, −5.5 to 2.8; FDR-adjusted *p* = 0.589]. After IPTW adjustment, remifentanil-based sedation was associated with shorter procedure duration [adjusted mean difference, −3.3 min; 95% CI, −5.1 to −1.4; FDR-adjusted *p* < 0.001], shorter anesthesia duration [adjusted mean difference, −3.6 min; 95% CI, −5.6 to −1.6; FDR-adjusted *p* < 0.001], and shorter PACU length of stay [adjusted mean difference, −4.9 min; 95% CI, −5.9 to −3.8; FDR-adjusted *p* < 0.001] (Table 4). Total propofol and midazolam doses were also lower in the remifentanil group after IPTW adjustment, and these differences remained significant after FDR correction. The total dose of the assigned opioid is provided descriptively and was not statistically compared because fentanyl and remifentanil doses are not directly pharmacologically equivalent (Table 4).

Recorded respiratory, hemodynamic, and PACU adverse outcomes are shown in Table 5. Respiratory events were relatively frequent in both groups. After IPTW adjustment, apnea was recorded in 25.9% of the fentanyl group and 18.8% of the remifentanil group [adjusted risk difference, −7.1 percentage points; 95% CI, −14.4 to 0.1; FDR-adjusted *p* = 0.179]. Airway maneuver requirement [26.2% vs. 18.8%; adjusted risk difference, −7.5 percentage points; 95% CI, −14.7 to −0.2; *p* = 0.043] and SpO_2_ < 90% events [26.2% vs. 17.6%; adjusted risk difference, −8.6 percentage points; 95% CI, −15.8 to −1.4; *p* = 0.019] were nominally lower in the remifentanil group, but these associations did not remain statistically significant after FDR correction. The adjusted risk difference for apnea was also numerically lower with remifentanil, but not statistically significant. Hemodynamic events, hemodynamic interventions, PACU nausea/vomiting, and PACU pain did not differ significantly between groups after IPTW adjustment and multiplicity correction (Table 5).

The association between remifentanil-based sedation and shorter postanesthetic recovery time was consistent across procedure types (Supplementary Table S1). After IPTW adjustment, recovery time was lower with remifentanil-based sedation in upper GI endoscopy, colonoscopy, and combined procedures. There was no evidence of effect modification by procedure type, with an opioid group × procedure type interaction *p*-value of 0.502. The primary finding was also supported by multivariable linear regression analysis (Supplementary Table S2). The multivariable-adjusted estimate was nearly identical to the main IPTW-adjusted estimate, showing a mean recovery time reduction of −11.3 s [95% CI, −13.2 to −9.4; *p* < 0.001] with remifentanil-based sedation.

## 4. Discussion

In this IPTW-adjusted single-center retrospective cohort study of adults undergoing routine GI endoscopy, remifentanil-based propofol sedation was associated with a shorter time to first purposeful response, shorter PACU length of stay, and lower cumulative propofol and midazolam exposure than fentanyl-based sedation. Documented MOAA/S scores and target-range sedation indicators were comparable between groups after IPTW adjustment, and lighter-than-target MOAA/S scores were not more frequent in the remifentanil group. These findings do not support a systematic difference in clinically assessed responsiveness-based sedation depth, although they cannot establish equivalent neurophysiologic hypnotic depth in the absence of processed electroencephalographic monitoring. Because this was a retrospective, nonrandomized study, the findings should be interpreted as associations reflecting the overall clinician-directed sedation strategy rather than isolated causal effects of remifentanil. After balancing 545 patients for measured baseline characteristics using stabilized IPTW, remifentanil-based sedation was associated with shorter procedure and anesthesia durations, reduced PACU length of stay, and lower cumulative propofol and midazolam requirements. Although apnea, airway-maneuver requirements, and SpO_2_ < 90% events were less frequent with remifentanil in nominal analyses, these differences were not significant after correction for multiple comparisons, while hemodynamic events, nausea or vomiting, and pain were similar between the groups. All sedation episodes were managed by the same institutional anesthesia team, and all procedures were performed by the same gastroenterology team. This structure likely reduced variability related to institutional practice, provider familiarity with remifentanil, airway-management thresholds, and endoscopist experience. Nevertheless, opioid choice and dose titration remained clinician-directed; therefore, individual clinician preference, anticipated procedural difficulty, real-time procedural conditions, or endoscopist-specific technique may still have influenced sedative dosing, procedural duration, and recovery outcomes.

The absolute difference in the primary outcome was modest. The adjusted mean reduction of 11.4 s reflects earlier return of responsiveness, defined as the first documented response to verbal command or purposeful awakening, and should not be interpreted as complete cognitive recovery, psychomotor recovery, or discharge readiness. Accordingly, the clinical relevance of this finding should be considered in the context of the broader recovery profile rather than the primary endpoint alone. In this cohort, the shorter time to first response was accompanied by shorter PACU length of stay and lower propofol and midazolam exposure, suggesting a consistent early recovery pattern. Nevertheless, whether an approximately 11 s difference in first purposeful response is clinically meaningful for individual patients remains uncertain and may be more relevant at the workflow level in high-volume endoscopy units. The shorter time to purposeful awakening observed in our study is consistent with the rapid and non-accumulative offset of remifentanil after discontinuation [16]. This pharmacokinetic advantage is particularly relevant to brief endoscopic procedures, in which residual opioid and hypnotic effects become apparent as soon as the procedural stimulation ceases. Randomized studies have similarly reported faster recovery with propofol-remifentanil than with midazolam-fentanyl-based sedation during colonoscopy [13,17], whereas remifentanil added to propofol shortened awakening and recovery after upper GI endoscopy [12]. Large observational data have also demonstrated longer PACU stays when fentanyl is added to propofol, indicating that residual fentanyl effects remain clinically relevant even after relatively short procedures [5]. However, the difference in recovery cannot be attributed to opioid kinetics alone.

Remifentanil provides potent and readily titratable analgesia, which may allow procedural tolerance with lower concomitant hypnotic exposure in some sedation strategies. In the present cohort, lower cumulative propofol and midazolam doses were observed in the remifentanil group; however, because opioid choice and sedative titration were clinician-directed, these differences should be interpreted as components of the overall sedation strategy rather than independent pharmacologic effects of remifentanil alone. This propofol-sparing effect has also been reported during upper GI endoscopy [18]. The lower cumulative propofol and midazolam exposure in our remifentanil group reduced the residual hypnotic burden and probably contributed to faster awakening. In contrast, Ho et al. found no recovery advantage with alfentanil over fentanyl during balanced propofol sedation [19]. Although alfentanil has a shorter duration of action than fentanyl, both drugs have a greater potential for residual effects than remifentanil does. Differences in hypnotic dosing, sedation depth, administration technique, and recovery definitions may also account for the absence of between-group differences. Moreover, faster awakening does not invariably translate into earlier discharge, which additionally depends on cognitive and functional recovery, post-procedural symptoms, institutional discharge criteria, and the local workflow [20]. Overall, the observed early recovery profile may reflect the combined contribution of remifentanil’s rapid offset and lower concomitant sedative exposure, but these associations cannot be separated from clinician-directed titration in this retrospective design.

The shorter PACU stay and lower cumulative propofol and midazolam exposure observed with remifentanil indicate that the recovery advantage extended beyond the initial return to responsiveness. However, the lower cumulative propofol and midazolam doses observed in the remifentanil group should be interpreted as components of the clinician-directed remifentanil-based sedation strategy rather than as independent pharmacologic effects of remifentanil alone. Clinicians selecting remifentanil may have anticipated its sedative-sparing potential and titrated propofol or midazolam more conservatively. Conversely, remifentanil’s rapid titratability and potent analgesic effect may have allowed procedural tolerance at the target MOAA/S range with lower concomitant hypnotic exposure. Nevertheless, MOAA/S is an intermittent responsiveness-based clinical scale and cannot exclude unmeasured differences in hypnotic depth between groups. Because opioid choice and subsequent sedative titration were not randomized or protocol-fixed, these possibilities cannot be separated in the present retrospective design. Similar improvements in recovery room efficiency have been reported with propofol-remifentanil regimens during colonoscopy [13,17]. The lower hypnotic requirement is consistent with the synergistic interaction between remifentanil and propofol, whereby rapidly titratable analgesia suppresses responses to procedural stimulation and permits adequate sedation with smaller doses of propofol [18]. In contrast, shorter procedure and anesthesia durations should not be interpreted as direct pharmacologic effects of opioid choice. These intervals may have been influenced by procedural complexity, endoscopist technique, patient movement, intraprocedural interruptions, workflow, and clinician dosing decisions.

A major concern limiting the routine use of remifentanil is its potential to cause abrupt respiratory depression, apnea, and hemodynamic deterioration, particularly when combined with propofol. In a randomized gastroscopy study, Xu et al. reported a higher incidence of apnea with remifentanil than with fentanyl, although hypoxemia and bag-mask ventilation did not differ between the groups [4]. Moerman et al. observed frequent hypoventilation during remifentanil sedation, whereas Mandel et al. found that the need for airway rescue varied substantially according to remifentanil delivery and sedation depth [14,21]. Conversely, carefully titrated propofol-remifentanil sedation has been associated with satisfactory hemodynamic stability and only minor respiratory depression [22]. Careful dose titration and continuous cardiorespiratory monitoring, including capnography, during deep propofol sedation remain essential [23]. In the present cohort, apnea, airway maneuver requirement, and SpO_2_ < 90% events were recorded relatively frequently in both groups. Although these respiratory outcomes were numerically lower in the remifentanil group, the differences did not remain statistically significant after FDR correction and should not be interpreted as evidence of respiratory safety superiority or safety equivalence. These findings suggest only that, within this anesthesiologist-directed and capnography-monitored sedation pathway, remifentanil was not associated with a statistically significant adjusted increase in recorded early respiratory or hemodynamic event occurrence. Because event-level details such as duration, severity, timing, escalation of care, and response to intervention were not consistently available, the safety findings should be interpreted cautiously as comparisons of documented event occurrence rather than as evidence of graded respiratory morbidity or safety equivalence.

The strengths of this study lie in the size and internal structure of the cohort, with one independent procedure per patient, detailed capture of drug exposure and recovery metrics, and consistent primary effect estimates across IPTW, log-transformed, multivariable, and procedure-specific analyses. The absence of extreme weights and the achievement of covariate balance after weighting strengthen the confidence that the observed association was not driven by measured baseline differences, while multiplicity correction limited the overinterpretation of secondary findings.

### Limitations

This study has several limitations. First, the retrospective, nonrandomized design precludes causal inference, and IPTW can balance only measured covariates. Although all cases were managed by the same fixed anesthesia and gastroenterology teams, residual confounding may persist due to clinician-directed opioid selection, individual clinician preference, anticipated procedural difficulty, endoscopist-specific technique, patient movement, transient sedation-depth fluctuations, and rescue-dosing patterns. Second, processed electroencephalographic monitoring was not routinely used; therefore, equivalence of neurophysiologic hypnotic depth could not be directly assessed. Sedation depth was clinically targeted and documented using MOAA/S, but MOAA/S is an intermittent responsiveness-based clinical scale and may not capture continuous fluctuations in hypnotic depth throughout the procedure. Third, propofol and midazolam doses and procedure duration were recorded after opioid selection and should therefore be regarded as components of the sedation strategy rather than independent causal effects. Fourth, recovery was defined by the first purposeful response and did not capture complete cognitive recovery, psychomotor recovery, or discharge readiness. Fifth, respiratory and hemodynamic outcomes were retrospectively extracted as recorded event occurrence only; event duration, severity, timing, escalation level, and response to intervention were not consistently available. Similarly, detailed time-stamped rescue-dosing trajectories and waveform-level capnography data were not uniformly available; therefore, dynamic dose–response or ventilation-pattern analyses could not be performed. Sixth, the study compared remifentanil- and fentanyl-based propofol sedation and did not include patients receiving propofol alone, midazolam alone, or other sedative-only strategies used in some institutions. Therefore, the findings cannot determine whether either opioid-adjunct strategy is superior or inferior to propofol-only or midazolam-only sedation approaches. Finally advanced, emergency, and higher-risk endoscopic procedures were excluded; thus, the findings should not be extrapolated to advanced endoscopic procedures, emergency endoscopy, patients requiring general anesthesia or advanced airway instrumentation, or procedures expected to require prolonged deep sedation. Future studies should use standardized dosing protocols, continuous ventilation and sedation-depth monitoring, validated cognitive and discharge endpoints, and formal workflow and cost analyses in multicenter prospective cohorts or randomized trials.

## 5. Conclusions

Remifentanil-based sedation was associated with a modest decrease in time to first purposeful response compared with fentanyl-based sedation in adults undergoing routine GI endoscopy. It was also associated with a shorter PACU length of stay and lower propofol and midazolam exposure, whereas adjusted respiratory and hemodynamic event rates did not differ significantly after multiplicity correction. Documented MOAA/S-based clinically assessed sedation depth was comparable between groups, and lighter-than-target sedation was not more frequent in the remifentanil group; however, MOAA/S cannot establish equivalent neurophysiologic hypnotic depth. These findings suggest a more favorable early recovery profile with remifentanil-based sedation under anesthesiologist-directed monitoring, but they should be interpreted as nonrandomized associations rather than evidence of independent causal effects or safety equivalence.

## Figures and Tables

**Table 1:** Baseline characteristics before inverse probability of treatment weighting.

Variable		All Patients (n = 545)	Fentanyl Group (n = 280)	Remifentanil Group (n = 265)	Difference [95% CI]	SMD	*p*-Value
Demographic characteristics							
Age, years		61 [49–69]	62 [49–70]	61 [49–69]	−1 [−3 to 1]	0.056	0.446
Sex, n (%)	Female	279 (51.2)	144 (51.4)	135 (50.9)	−0.5 pp [−8.8 to 7.9]	0.010	0.910
	Male	266 (48.8)	136 (48.6)	130 (49.1)	+0.5 pp [−7.9 to 8.8]		
ASA physical status, n (%)	I	47 (8.6)	25 (8.9)	22 (8.3)	−0.6 pp [−5.4 to 4.2]	0.046	0.954
	II	357 (65.5)	184 (65.7)	173 (65.3)	−0.4 pp [−8.4 to 7.5]		
	III	138 (25.3)	69 (24.6)	69 (26.0)	+1.4 pp [−5.9 to 8.7]		
	IV	3 (0.6)	2 (0.7)	1 (0.4)	−0.3 pp [−2.2 to 1.5]		
Procedure type, n (%)							
Upper gastrointestinal endoscopy		190 (34.9)	89 (31.8)	101 (38.1)	+6.3 pp [−1.7 to 14.2]	0.133	0.300
Colonoscopy		183 (33.6)	98 (35.0)	85 (32.1)	−2.9 pp [−10.8 to 5.0]		
Combined upper gastrointestinal endoscopy and colonoscopy		172 (31.6)	93 (33.2)	79 (29.8)	−3.4 pp [−11.1 to 4.4]		
Comorbidities and medication history							
Smoking history, n (%)		158 (29.0)	84 (30.0)	74 (27.9)	−2.1 pp [−9.6 to 5.5]	0.046	0.594
Suspected/diagnosed OSAS, n (%)		6 (1.1)	3 (1.1)	3 (1.1)	+0.1 pp [−2.1 to 2.3]	0.006	1.000
COPD/asthma, n (%)		53 (9.7)	34 (12.1)	19 (7.2)	−5.0 pp [−10.0 to 0.0]	0.169	0.050
Cardiovascular disease, n (%)		113 (20.7)	60 (21.4)	53 (20.0)	−1.4 pp [−8.2 to 5.4]	0.035	0.681
Hypertension, n (%)		233 (42.8)	133 (47.5)	100 (37.7)	−9.8 pp [−17.9 to −1.5]	0.198	0.021
Diabetes mellitus, n (%)		130 (23.9)	70 (25.0)	60 (22.6)	−2.4 pp [−9.5 to 4.8]	0.055	0.518
Chronic kidney disease, n (%)		21 (3.9)	14 (5.0)	7 (2.6)	−2.4 pp [−5.8 to 1.0]	0.123	0.153
Chronic liver disease, n (%)		30 (5.5)	11 (3.9)	19 (7.2)	+3.2 pp [−0.7 to 7.4]	0.142	0.097
Neurological disease, n (%)		43 (7.9)	18 (6.4)	25 (9.4)	+3.0 pp [−1.6 to 7.7]	0.111	0.193
Anticoagulant/antiplatelet therapy, n (%)		133 (24.4)	72 (25.7)	61 (23.0)	−2.7 pp [−9.8 to 4.5]	0.063	0.464

Abbreviations: ASA, American Society of Anesthesiologists; CI, confidence interval; COPD, chronic obstructive pulmonary disease; OSAS, obstructive sleep apnea syndrome; pp, percentage points; SMD, standardized mean difference. Note: Data are presented as median [25th–75th percentile], or n (%), as appropriate. Non-normally distributed or ordinal variables were compared using the Mann–Whitney U test. Categorical variables were compared using the chi-square test or Fisher’s exact test, as appropriate. Differences were reported as Hodges–Lehmann median differences or absolute percentage-point differences with 95% CIs. For multi-level categorical variables, CIs represent level-specific one-versus-rest differences, whereas *p*-values represent the global comparison across all levels. SMDs were reported as unweighted absolute standardized mean differences before IPTW; values < 0.10 were considered indicative of a negligible imbalance. All differences were expressed as remifentanil minus fentanyl. All *p*-values were two-sided, and values below 0.05 were considered statistically significant.

**Table 2:** Covariate balance before and after inverse probability of treatment weighting.

Variable		IPTW-Weighted Fentanyl Group (n = 280)	IPTW-Weighted Remifentanil Group (n = 265)	Unweighted SMD	IPTW-Weighted SMD
Demographic characteristics					
Age, years		61 [48–69]	61 [49–70]	0.056	0.009
Sex, n (%)	Female	144.7 (51.6)	137.7 (51.8)	0.010	0.004
	Male	135.8 (48.4)	128.1 (48.2)		
ASA physical status, n (%)	I	24.0 (8.5)	22.6 (8.5)	0.046	0.010
	II	184.2 (65.7)	173.2 (65.2)		
	III	70.7 (25.2)	68.2 (25.7)		
	IV	1.6 (0.6)	1.7 (0.7)		
Procedure type, n (%)					
Upper gastrointestinal endoscopy		99.2 (35.4)	92.1 (34.7)	0.133	0.019
Colonoscopy		93.8 (33.5)	91.3 (34.3)		
Combined upper gastrointestinal endoscopy and colonoscopy		87.4 (31.2)	82.4 (31.0)		
Comorbidities and medication history					
Smoking history, n (%)		80.5 (28.7)	77.5 (29.1)	0.046	0.010
Suspected/diagnosed OSAS, n (%)		3.5 (1.2)	3.0 (1.1)	0.006	0.012
COPD/asthma, n (%)		27.2 (9.7)	27.4 (10.3)	0.169	0.021
Cardiovascular disease, n (%)		57.8 (20.6)	54.4 (20.5)	0.035	0.003
Hypertension, n (%)		118.9 (42.4)	114.0 (42.9)	0.198	0.010
Diabetes mellitus, n (%)		68.7 (24.5)	65.8 (24.8)	0.055	0.006
Chronic kidney disease, n (%)		10.9 (3.9)	11.0 (4.2)	0.123	0.014
Chronic liver disease, n (%)		17.1 (6.1)	15.0 (5.6)	0.142	0.020
Neurological disease, n (%)		20.9 (7.5)	20.2 (7.6)	0.111	0.006
Anticoagulant/antiplatelet therapy, n (%)		68.0 (24.2)	65.8 (24.8)	0.063	0.012

Abbreviations: ASA, American Society of Anesthesiologists; COPD, chronic obstructive pulmonary disease; IPTW, inverse probability of treatment weighting; OSAS, obstructive sleep apnea syndrome; SMD, standardized mean difference. Note: The propensity score model estimated the probability of receiving remifentanil-based sedation using age, sex, ASA physical status, procedure type, smoking history, suspected/diagnosed OSAS, COPD/asthma, cardiovascular disease, hypertension, diabetes mellitus, chronic kidney disease, chronic liver disease, neurological disease, and anticoagulant/antiplatelet therapy. The stabilized IPTWs were calculated for the average treatment effect. Weighted categorical variables are presented as weighted n (%), and weighted age is presented as weighted median [25th-75th percentile]. SMDs were reported before and after IPTW; for multi-level categorical variables, the SMD represents the largest absolute level-specific SMD across categories. Absolute SMD values < 0.10 were considered indicative of an acceptable balance. Each patient contributed to one procedure; therefore, patients and procedures were numerically equivalent in the analysis. All 545 independent observations were retained for IPTW analysis. The effective sample size after weighting was 267.6 in the fentanyl group and 249.5 in the remifentanil group.

**Table 3:** Primary outcome analysis of postanesthetic recovery time.

Analysis	Fentanyl Group (n = 280)	Remifentanil Group (n = 265)	Effect Estimate [95% CI]	*p*-Value
Crude recovery time, s	33.8 ± 10.2	22.5 ± 11.5	−11.3 [−13.1 to −9.4]	<0.001
IPTW-weighted recovery time, s	34.1 [32.8 to 35.3]	22.6 [21.2 to 24.0]		
IPTW-adjusted mean difference, s			−11.4 [−13.4 to −9.5]	<0.001
IPTW log-transformed sensitivity analysis, geometric mean ratio			0.628 [0.591 to 0.667]	<0.001

Abbreviations: CI, confidence interval; IPTW, inverse probability of treatment weighting. Note: The primary outcome was postanesthetic recovery time, defined as the time from the end of the procedure to the response to verbal command or purposeful awakening, measured in seconds. This endpoint reflects early return of responsiveness and does not represent complete cognitive recovery, psychomotor recovery, or discharge readiness. The crude recovery time is presented as mean ± standard deviation and was compared using the Welch independent-samples t-test. The IPTW-weighted recovery time is presented as a weighted mean [95% CI]. The IPTW-adjusted effect estimate was obtained using a weighted generalized linear model with stabilized IPTWs and reported as the mean difference. The log-transformed IPTW sensitivity analysis is reported as the geometric mean ratio of remifentanil to fentanyl. Absolute effect estimates were expressed as remifentanil minus fentanyl; negative values indicated a shorter recovery time in the remifentanil group. A geometric mean ratio below 1 indicated a shorter recovery time in the remifentanil group on the relative scale. All *p*-values were two-sided, and values below 0.05 were considered statistically significant.

**Table 4:** Documented sedation depth, secondary procedural time outcomes, and anesthetic drug exposure.

Outcome	Crude Fentanyl Group (n = 280)	Crude Remifentanil Group (n = 265)	Crude Effect Estimate [95% CI]	Crude *p*-Value	IPTW-Weighted Fentanyl Group (n = 280)	IPTW-Weighted Remifentanil Group (n = 265)	IPTW-Adjusted Effect Estimate [95% CI]	IPTW *p*-Value	FDR-Adjusted *p*-Value
MOAA/S score	2 [2-2]	2 [2-2]	0.0 [0.0 to 0.0]	0.288	2 [2-2]	2 [2-2]	0.0 [0.0 to 0.0]	0.209	0.314
Target MOAA/S range 2–3, n (%)	263 (93.9)	246 (92.8)	−1.1 pp [−5.5 to 3.2]	0.606	264.4 (94.2)	246.9 (92.9)	−1.3 pp [−5.5 to 2.8]	0.523	0.589
MOAA/S ≤ 1, deeper than target, n (%)	10 (3.6)	12 (4.5)	1.0 pp [−2.5 to 4.6]	0.571	9.5 (3.4)	12.1 (4.6)	1.2 pp [−2.1 to 4.5]	0.486	0.589
MOAA/S ≥ 4, lighter than target, n (%)	7 (2.5)	7 (2.6)	0.1 pp [−2.8 to 3.1]	0.917	6.6 (2.4)	6.7 (2.5)	0.2 pp [−2.4 to 2.7]	0.898	0.898
Procedure duration, min	25.4 ± 12.1	21.5 ± 10.2	−3.9 [−5.8 to −2.0]	<0.001	25.1 [23.7 to 26.4]	21.8 [20.5 to 23.1]	−3.3 [−5.1 to −1.4]	<0.001	0.001
Anesthesia duration, min	27.8 ± 12.6	23.6 ± 10.8	−4.2 [−6.2 to −2.2]	<0.001	27.5 [26.1 to 28.9]	23.9 [22.5 to 25.3]	−3.6 [−5.6 to −1.6]	<0.001	0.001
PACU length of stay, min	24.6 ± 6.5	19.8 ± 5.5	−4.7 [−5.7 to −3.7]	<0.001	24.7 [23.9 to 25.5]	19.8 [19.2 to 20.5]	−4.9 [−5.9 to −3.8]	<0.001	<0.001
Total propofol dose, mg	151.6 ± 75.1	119.7 ± 66.3	−31.9 [−43.8 to −20.0]	<0.001	150.5 [141.3 to 159.8]	120.9 [112.4 to 129.4]	−29.7 [−42.2 to −17.1]	<0.001	<0.001
Total midazolam dose, mg	1.4 ± 0.7	1.0 ± 0.6	−0.5 [−0.6 to −0.3]	<0.001	1.4 [1.3 to 1.5]	1.0 [0.9 to 1.0]	−0.4 [−0.6 to −0.3]	<0.001	<0.001
Total assigned opioid dose, μg	53.5 ± 15.1	47.7 ± 16.1	-	-	53.9 [52.0 to 55.8]	47.6 [45.6 to 49.6]	-	-	-

Abbreviations: CI, confidence interval; FDR, false discovery rate; IPTW, inverse probability of treatment weighting; MOAA/S, Modified Observer’s Assessment of Alertness/Sedation; PACU, post-anesthesia care unit; μg, microgram. Note: Crude continuous variables are presented as mean ± standard deviation and were compared using the Welch independent-samples t-test. MOAA/S score is presented as median [25th–75th percentile] and was compared using the Mann–Whitney U test. Binary sedation-depth indicators are presented as n (%) and were compared using the chi-square test or Fisher’s exact test, as appropriate. IPTW-weighted continuous variables are presented as weighted mean [95% CI], IPTW-weighted MOAA/S score as weighted median [25th–75th percentile], and IPTW-weighted binary indicators as weighted n (%). IPTW-adjusted effect estimates were obtained using weighted linear models for continuous outcomes and weighted linear probability models for binary outcomes, with stabilized IPTWs and robust standard errors. FDR-adjusted IPTW *p*-values were calculated using the Benjamini–Hochberg method across the secondary procedural/sedation-depth/drug-exposure outcome family. The total assigned opioid dose was excluded from inferential testing and multiplicity adjustment because it was reported descriptively only, and was not statistically compared because fentanyl and remifentanil doses are not directly pharmacologically equivalent. All effect estimates are expressed as remifentanil minus fentanyl; negative values indicate lower values in the remifentanil group.

**Table 5:** Periprocedural adverse events and safety outcomes.

Outcome	Crude Fentanyl Group (n = 280)	Crude Remifentanil Group (n = 265)	Crude Risk Difference [95% CI]	Crude *p*-Value	IPTW-Weighted Fentanyl Group (n = 280)	IPTW-Weighted Remifentanil Group (n = 265)	IPTW-Adjusted Risk Difference [95% CI]	IPTW *p*-Value	FDR-Adjusted *p*-Value
Apnea event, n (%)	71 (25.4)	48 (18.1)	−7.2 pp [−14.1 to −0.3]	0.041	72.6 (25.9)	49.9 (18.8)	−7.1 pp [−14.4 to 0.1]	0.054	0.179
Airway maneuver requirement, n (%)	72 (25.7)	48 (18.1)	−7.6 pp [−14.4 to −0.6]	0.032	73.6 (26.2)	49.9 (18.8)	−7.5 pp [−14.7 to −0.2]	0.043	0.179
SpO_2_ < 90% event, n (%)	72 (25.7)	45 (17.0)	−8.7 pp [−15.5 to −1.8]	0.013	73.6 (26.2)	46.8 (17.6)	−8.6 pp [−15.8 to −1.4]	0.019	0.179
Composite hemodynamic event/intervention, n (%)	27 (9.6)	25 (9.4)	−0.2 pp [−5.2 to 4.8]	0.934	26.5 (9.4)	24.1 (9.1)	−0.4 pp [−5.3 to 4.5]	0.880	0.880
Hypotension event, n (%)	25 (8.9)	21 (7.9)	−1.0 pp [−5.8 to 3.8]	0.673	24.5 (8.7)	20.2 (7.6)	−1.1 pp [−5.8 to 3.5]	0.630	0.794
Bradycardia event, n (%)	4 (1.4)	6 (2.3)	+0.8 pp [−1.7 to 3.6]	0.535	4.1 (1.4)	6.2 (2.3)	+0.9 pp [−1.5 to 3.3]	0.460	0.767
Vasopressor use, n (%)	4 (1.4)	5 (1.9)	+0.5 pp [−2.0 to 3.1]	0.746	4.1 (1.5)	4.9 (1.9)	+0.4 pp [−1.8 to 2.6]	0.738	0.820
Fluid bolus use, n (%)	20 (7.1)	16 (6.0)	−1.1 pp [−5.4 to 3.2]	0.604	19.4 (6.9)	15.7 (5.9)	−1.0 pp [−5.2 to 3.2]	0.635	0.794
PACU nausea/vomiting, n (%)	0 (0.0)	1 (0.4)	+0.4 pp [−1.0 to 2.1]	0.486	0.0 (0.0)	1.1 (0.4)	+0.4 pp [−0.4 to 1.3]	0.317	0.634
PACU pain, n (%)	2 (0.7)	0 (0.0)	−0.7 pp [−2.6 to 0.8]	0.499	2.1 (0.8)	0.0 (0.0)	−0.8 pp [−1.8 to 0.3]	0.169	0.423

Abbreviations: CI, confidence interval; FDR, false discovery rate; IPTW, inverse probability of treatment weighting; PACU, post-anesthesia care unit; pp, percentage points; SpO_2_, peripheral oxygen saturation. Note: Crude values were presented as n (%) and were compared using the chi-square test or Fisher’s exact test. Crude risk differences were reported with 95% CIs using the Newcombe–Wilson method. IPTW-weighted values are presented as weighted n (%); weighted n values are pseudo-counts derived from the stabilized IPTWs. IPTW-adjusted risk differences were estimated using weighted linear probability models with stabilized IPTWs and robust, survey-based standard errors. FDR-adjusted IPTW *p*-values were calculated using the Benjamini–Hochberg method across the binary safety outcomes. Respiratory and hemodynamic events were not mutually exclusive; because each patient contributed to one procedure, an individual observation could contribute to more than one event category. The composite hemodynamic event/intervention was defined as the occurrence of hypotension, bradycardia, vasopressor use, or fluid bolus administration. These outcomes represent recorded event occurrence only; duration, severity, exact timing, escalation level, and response to intervention were not consistently available for retrospective analysis. All risk differences were expressed as remifentanil minus fentanyl; negative values indicated a lower event frequency in the remifentanil group.

## Data Availability

The de-identified data supporting the findings of this study are available from the corresponding author upon reasonable request, subject to institutional and ethics committee restrictions.

## Supplementary Materials

The following supporting information can be downloaded at https://www.mdpi.com/article/10.3390/jcm15187276/s1. Supplementary Figure S1. Propensity score overlap and stabilized IPTW distribution; Supplementary Table S1. Subgroup analysis of postanesthetic recovery time according to procedure type; Supplementary Table S2. Sensitivity analysis of postanesthetic recovery time using multivariable linear regression.

## Abbreviations

The following abbreviations are used in this manuscript:

ASA	American Society of Anesthesiologists
CI	Confidence interval
COPD	Chronic obstructive pulmonary disease
FDR	False discovery rate
GI	Gastrointestinal
IPTW	Inverse probability of treatment weighting
MOAA/S	Modified Observer’s Assessment of Alertness/Sedation
OSAS	Obstructive sleep apnea syndrome
PACU	Post-anesthesia care unit
pp	Percentage point
SD	Standard deviation
SMD	Standardized mean difference
SpO_2_	Peripheral oxygen saturation
STROBE	Strengthening the Reporting of Observational Studies in Epidemiology
