# Supplementary material for: Recovery Profiles After MOAA/S-Guided Remifentanil- Versus Fentanyl-Based Propofol Sedation for Gastrointestinal Endoscopy: An IPTW-Adjusted Retrospective Cohort Study
Source: J Clin Med. 2026 Sep 19;15(18):7276. doi: 10.3390/jcm15187276 (PMC13608613; doi:10.3390/jcm15187276)
Supplement: Supplementary file 1 [file jcm-15-07276-s001.zip › jcm-4527065-supplementary.pdf]

Supplementary Table S1. Subgroup analysis of postanesthetic recovery time according to procedure type.

Procedure Type	Fentanyl Group (n = 280)	Remifentanil Group (n = 265)	IPTW-Weighted Fentanyl Group	IPTW-Weighted Remifentanil group	IPTW-Adjusted Mean Difference [95% CI]	*p*-Value	*p* for Interaction
Upper gastrointestinal endoscopy	89 (31.8)	101 (38.1)	34.3 [31.8 to 36.8]	21.3 [19.2 to 23.4]	-13.0 [-16.3 to -9.7]	<0.001	0.502
Colonoscopy	98 (35.0)	85 (32.1)	32.7 [31.0 to 34.3]	21.9 [19.6 to 24.1]	-10.8 [-13.6 to -8.0]	<0.001	
Combined upper gastrointestinal endoscopy and colonoscopy	93 (33.2)	79 (29.8)	35.3 [32.9 to 37.6]	24.9 [21.9 to 27.9]	-10.4 [-14.2 to -6.5]	<0.001	

Abbreviations: CI, confidence interval; IPTW, inverse probability of treatment weighting. Note: The outcome was the postanesthetic recovery time, measured in seconds. Procedure-type subgroup analyses were performed using the stabilized IPTW weights. Values are presented as IPTW-weighted means [95% CI]. The percentages were calculated within each opioid group. Each patient contributed to one procedure; therefore, the subgroup counts represent both patients and procedures. The *p* value for interaction was obtained from an IPTW-weighted linear model including the opioid group, procedure type, and their interaction. Effect estimates were expressed as remifentanil minus fentanyl; negative values indicated a shorter recovery time in the remifentanil group.

Supplementary Table S2. Sensitivity analysis of postanesthetic recovery time using multivariable linear regression.

Analysis	Fentanyl Group (n = 280)	Remifentanil Group (n = 265)	Effect Estimate [95% CI]	*p*-Value
Crude comparison	33.8 ± 10.2	22.5 ± 11.5	-11.3 [-13.1 to -9.4]	<0.001
Main IPTW-adjusted analysis	34.1 [32.8 to 35.3]	22.6 [21.2 to 24.0]	-11.4 [-13.4 to -9.5]	<0.001
Multivariable-adjusted linear regression	-	-	-11.3 [-13.2 to -9.4]	<0.001

Abbreviations: ASA, American Society of Anesthesiologists; CI, confidence interval; IPTW, inverse probability of treatment weighting; OSAS, obstructive sleep apnea syndrome. Note: The outcome was postanesthetic recovery time, measured in seconds. Crude values are presented as mean ± standard deviation and were compared using the Welch independent-samples t-test. The main IPTW-adjusted estimate was obtained using a weighted generalized linear model with stabilized IPTW weight. The multivariable-adjusted sensitivity model used conventional linear regression with HC3 robust standard errors and included age, sex, ASA physical status, procedure type, smoking history, suspected/diagnosed OSAS, chronic pulmonary disease, cardiovascular disease, hypertension, diabetes mellitus, chronic kidney disease, chronic liver disease, neurological disease, and anticoagulant/antiplatelet therapy. Effect estimates were expressed as remifentanil minus fentanyl; negative values indicated a shorter recovery time in the remifentanil group.

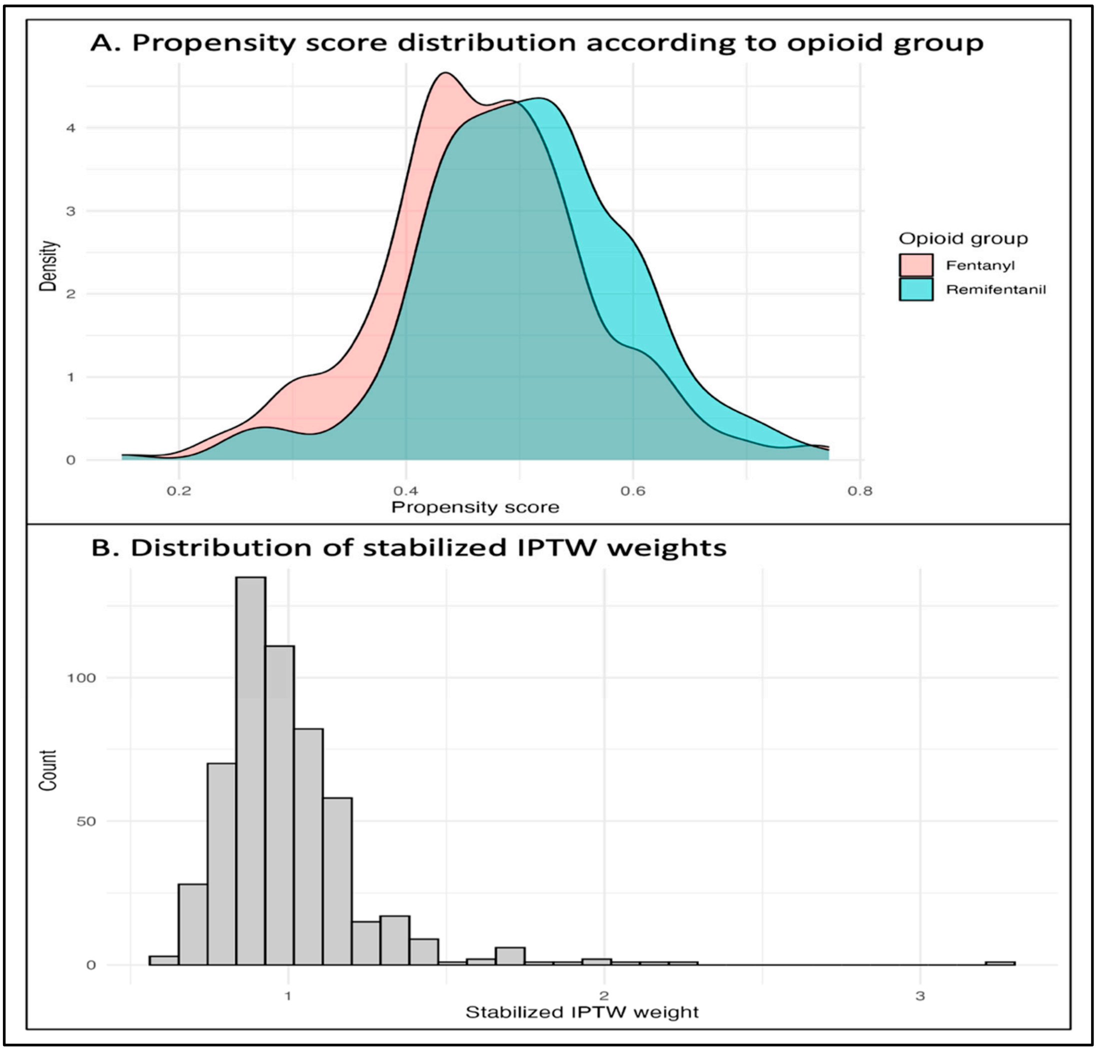

Supplementary Figure S1. Propensity score overlap and stabilized IPTW distribution. Abbreviations: IPTW, inverse probability of treatment weighting. A. Propensity score distribution according to the opioid group. Adequate overlap between the fentanyl and remifentanil groups was observed across the main propensity score range. B. Distribution of the stabilized IPTW weights. The weights were concentrated around 1, with no stabilized weight exceeding 5, indicating no evidence of influential or extreme weights.
